# Forecasting the outcome of a time-varying Bernoulli process: Data from a laboratory experiment

**DOI:** 10.1016/j.dib.2017.10.007

**Published:** 2017-10-06

**Authors:** Mel W. Khaw, Luminita Stevens, Michael Woodford

**Affiliations:** a Department of Economics, Columbia University, 10027 New York, NY, USA; b Department of Economics, University of Maryland, 20743 College Park, MD, USA

## Abstract

The data presented in this article are related to the research article entitled “Discrete Adjustment to a Changing Environment: Experimental Evidence” (Khaw et al., 2017) [1]. We present data from a laboratory experiment that asks subjects to forecast the outcome of a time-varying Bernoulli process. On a computer program, subjects draw rings with replacement from a virtual box containing green and red rings in an unknown proportion. Subjects provide their estimates of the probability of drawing a green ring. They are rewarded for their participation and for the accuracy of their estimates. The actual probability of drawing a green ring is initially drawn from a uniform distribution. It then changes intermittently throughout the session, and each subsequent probability is an independent draw from the uniform distribution. Each session involves 1000 ring draws. The dataset contains the values of the underlying probability, the sequence of ring draws that are realized, and the subjects’ estimates and response times. The dataset contains the performance of 11 subjects who each completed 10 sessions over the course of several days.

**Specifications Table**

**Table:** 

Subject area	*Economics, Psychology*
More specific subject area	*Behavioral Economics, Experimental Economics, Cognitive Psychology*
Type of data	*Text file*
How data was acquired	*Computer program and human subjects*
Data format	*Raw*
Experimental factors	*N/A*
Experimental features	*Probability estimation of stochastic observations of a changing variable*
Data source location	*N/A*
Data accessibility	*With article*

**Value of the data**

• These data are valuable for analyzing how people form and update forecasts.
• The experiment defines the subjects’ objective function and rewards subjects for their performance, thus researchers can treat the decision-maker's objective as known.
• The experiment controls what information subjects have about the task at each point in time, enabling researchers to test hypotheses about how subjects process the information in real time.
• Subjects control how quickly to perform the task and how often to update their estimates, hence the data can be used for studies of response time, the nature of adjustment dynamics, and the relationship between accuracy and speed in performing a task.
• The experiment yields a large number of observations per subject, allowing for analysis both within and across subjects.

## 1 Data

The dataset contains the outcomes of a laboratory experiment [1] on probability estimation. The data are the realizations of a Bernoulli random variable that governs the probability of drawing a green ring out of a box with green and red rings; the underlying probabilities of drawing a green ring, which change intermittently over the course of each session; and our subjects’ estimates of these probabilities and their response times (the elapsed time in between subjects’ clicks of the ‘NEXT’ button, which we describe further below). There are 109,890 observations corresponding to 11 subjects, 10 sessions per subject, and 999 observations per session (discarding, for each trial, the last estimate, which was not scored). Out of 110 sessions, 91 had unique sequences of ring realizations and 19 were repetitions of one of these sequences. The repeated sessions occurred randomly across the subjects. The subjects were undergraduate and Master's students at Columbia University, and the experiment took place on campus. Each session consisted of a short practice session followed by the actual session of 1000 ring draws. Each session lasted approximately 27 minutes.

## 2 Experimental design, materials and methods

### 2.1 Computer interface

**Fig. 1:**
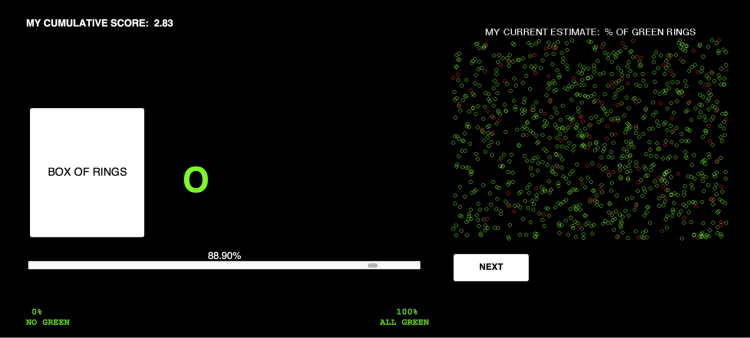
Screenshot of the experiment's user interface.

On a computer program, subjects are presented with the interface shown in Fig. 1. The screen displays a white box of rings that contains a hidden mix of red and green rings. Subjects report their forecast of the probability p of drawing a green ring from this box, using a mouse to move the slider bar at the bottom of the screen. The subject's current estimate $\hat{p}$ is displayed in percentage points, on top of the bar, and visually, in a box on the right side of the screen that displays a mix of 1000 green and red rings that reflects the probability of green rings indicated by the subject's slider position. Whenever the subject moves the slider, both the number on top of the bar and the box on the right adjust in real time. The top left corner of the interface displays the subject's current score. This interface is adapted from the experimental paradigm reported by Gallistel et al. [2].

Every session begins with the slider set at 0% and without any rings displayed on the interface. The subject begins with a guess and adjusts the position of the slider to indicate his or her estimate. When the subject is satisfied with her current estimate, she can click on the button “NEXT” at which point a new ring is drawn randomly from the box and displayed on the screen. Only the latest ring is displayed on the screen at any given moment. This process is repeated until the session ends (after 1000 ring draws).

### 2.2 Reward

The subject's reward is given by the quadratic scoring rule $r\left({\overset{ˆ}{p}}_{t};s_{t}\right)=1-{\left(s_{t}-{\overset{ˆ}{p}}_{t}\right)}^{2},$ where ${\overset{ˆ}{p}}_{t}$ is the slider position set by the subject and $s_{t}$ is the ring draw, equal to 1 for a green ring and 0 for a red ring. The total score is the sum of the rewards for all trials in a session. For each session, subjects received a fixed payment of $10 plus a variable payment, equal to $2 cents multiplied by the subject's total score.

### 2.3 Data generating process

The initial probability *p* of drawing a green ring from the box is drawn from the uniform distribution on the unit interval. After each ring draw, there is a constant probability equal to 0.5% of a box change. If there is a box change, the new probability of drawing a green ring is also an independent draw from the uniform distribution on the unit interval. The subjects are not told when a change in the box occurs, but they are told in advance that the box might change, and they know the probability of a regime change and the distribution from which the new box is drawn. Ricci and Gallistel [3] consider a similar experimental design, but one in which the probability is continuously changing. Before the start of each session, we explained to the subjects the reward function, the data generating process, and the optimal decision rule, using both written and verbal instructions.

### 2.4 Summary of the data

**Fig. 2:**
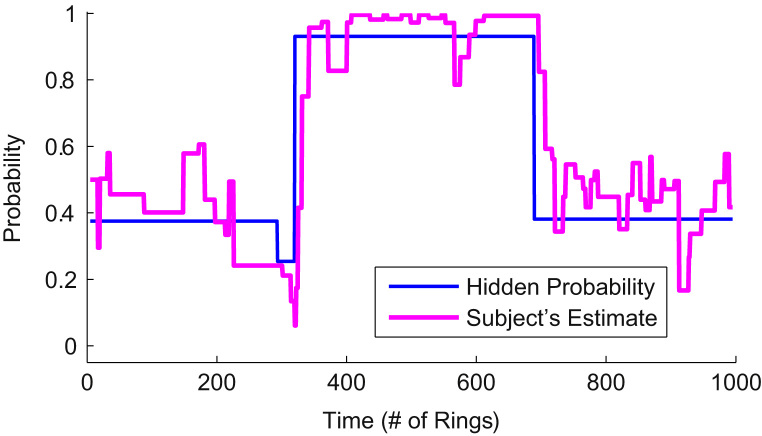
Example timeline of subject's estimate of the actual probability (Subject 10, Session 8).

Fig. 2 shows the outcomes of session 8 for Subject 10. The blue line shows the true probability of drawing a green ring from the box, and the magenta line shows the subject's estimate over the course of this session.

**Fig. 3:**
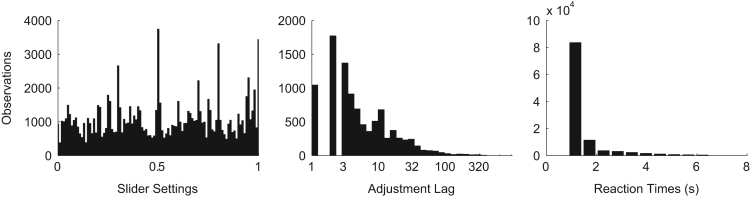
Distribution of observed estimates reported on the slider (left), observations collected before adjustment (middle), and reaction times (right).

Fig. 3 shows the distribution of estimates, adjustment lags, and reaction times, which measure the seconds between ring draws.

**Fig. 4:**
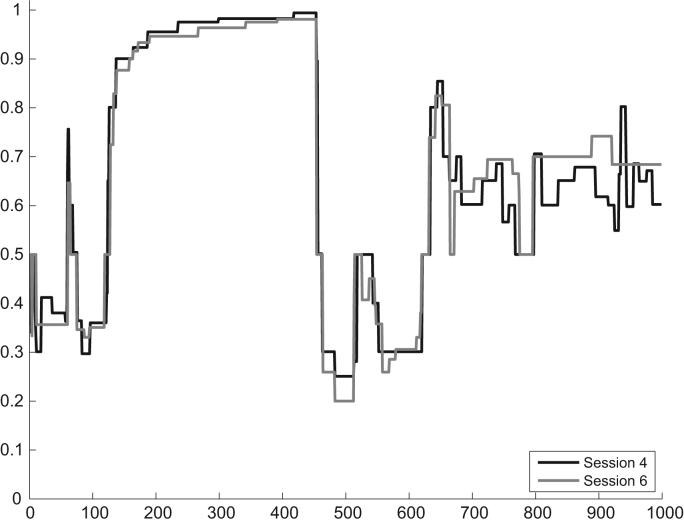
Example data using the repeated sequence of probabilities and ring draws (Subject 2, Session 4 and 6).

Fig. 4 shows the outcomes for Subject 2's repeated sessions. This subject saw the same sequence of rings during sessions 4 and 6. The figure shows the subject's reported estimates on these two occasions.
