# Supplementary material for: Forecasting the outcome of a time-varying Bernoulli process: Data from a laboratory experiment
Source: Data Brief. 2017 Oct 6;15:469–73. doi: 10.1016/j.dib.2017.10.007 (PMC5647523; doi:10.1016/j.dib.2017.10.007)
Supplement: Supplementary file 1 — Transparency document [file mmc1.docx]

The authors have no conflicts of interest to disclose
